# Multiple host-switching of Haemosporidia parasites in bats

**DOI:** 10.1186/1475-2875-6-157

**Published:** 2007-11-29

**Authors:** Linda Duval, Vincent Robert, Gabor Csorba, Alexandre Hassanin, Milijaona Randrianarivelojosia, Joe Walston, Thy Nhim, Steve M Goodman, Frédéric Ariey

**Affiliations:** 1 Muséum National d'Histoire Naturelle, USM 504 et UMR 5202, 55-61 rue Buffon, 75231 Paris Cedex 05, France; 2 Institut Pasteur du Cambodge, Unité Epidémiologie moléculaire, 5, Boulevard Monivong, BP 983, Phnom Penh, Cambodia; 3 Institut de Recherche pour le Développement, UR 77, 213 rue La Fayette, 75480 Paris Cedex 10, France; 4 Hungarian Natural History Museum, Department of Zoology, H-1083 Budapest, Ludovika tér 2, Hungary; 5 Institut Pasteur de Madagascar, Unité Paludisme, BP 1274, Antananarivo 101, Madagascar; 6 Wildlife Conservation Society, Cambodia Program, #21, St 21 Sangkat Tonlé Bassac, Khan Chamkarmorn, Phnom Penh, Cambodia; 7 Phnom Tamao Zoo and Wildlife Rescue Center, Department of Forestry and Wildlife, 40 Norodom Boulevard, Phnom Penh, Cambodia; 8 Care for Confiscated Wild Life, WildAid, St 99, Phnom Penh, Cambodia; 9 Field Museum of Natural History, 1400 South Lake Shore Drive, Chicago, Illinois 60605, USA; 10 WWF, BP 738, Antananarivo 101, Madagascar

## Abstract

**Background:**

There have been reported cases of host-switching in avian and lizard species of *Plasmodium *(Apicomplexa, Haemosporidia), as well as in those infecting different primate species. However, no evidence has previously been found for host-swapping between wild birds and mammals.

**Methods:**

This paper presents the results of the sampling of blood parasites of wild-captured bats from Madagascar and Cambodia. The presence of Haemosporidia infection in these animals is confirmed and cytochrome *b *gene sequences were used to construct a phylogenetic analysis.

**Results:**

Results reveal at least three different and independent Haemosporidia evolutionary histories in three different bat lineages from Madagascar and Cambodia.

**Conclusion:**

Phylogenetic analysis strongly suggests multiple host-switching of Haemosporidia parasites in bats with those from avian and primate hosts.

## Background

*Plasmodium falciparum *(Apicomplexa, Haemosporidia), the most dangerous of human malaria parasites, is responsible for at least one million deaths a year [1]. It has been suggested that its exceptional virulence, compared to the three other species of human *Plasmodium*, is due to its relatively recent host-shift from birds to humans and the short period for the latter to adapt to the parasite [2]. Given the heavy burden of *P. falciparum *on human populations around the tropics [3], there is a critical need to better understand the origin and evolution of this parasite and related organisms. *Plasmodium falciparum *belongs to a group that also infects a considerable range of birds, squamates, crocodilians, chelonians and non-human mammals [4]. These parasites are known to be virulent, invasive pathogens in a variety of wild animals and contribute to the parasite burden of natural populations, including several threatened species [5]. Host switching by these parasites could be the trigger for emerging virulent diseases [6-8].

Madagascar and Cambodia are two biodiversity "hot spots" [9]. The faunas of these areas, which are, in evolutionary terms, distant from one another, provide an attractive system for characterizing haemosporidian parasite species [10,11] and for evaluating host-parasite co-evolution and exchange. Madagascar was part to the Gondwana continent and was separated 160 million and 90 million years ago from Africa and from India, respectively; whereas, Cambodia originated from the Laurasia continent.

The paper presents the result of the screening of over 500 bats belonging to seven families from different field sites in Madagascar and Cambodia. Haemosporidia parasites were isolated in the bat families, Hipposideridae, Vespertilionidae and Megadermatidae. Molecular sequences of bat haemosporidian parasites were not previously available. Herein, the phylogenetic analyses with cytochrome *b *mitochondrial gene sequences illustrate the first documented example of a cross-class host-switching of haemosporidian parasites between birds and mammals.

## Methods

### Field sites and sample collection

Screened material was obtained in Madagascar and Cambodia. In Madagascar, specimens were obtained at eight different field sites in the provinces of Mahajanga, Toliara and Antananarivo. The fieldwork was conducted between November 2001 and late 2004, in collaboration with the Institut Pasteur de Madagascar and WWF-Madagascar. In Cambodia, two field sites were sampled in the provinces of Kampot and Mondolkiri between December 2004 and May 2006, in collaboration with the Institut Pasteur du Cambodia and Wildlife Conservation Society. The field research was conducted with local and national permits from the Direction des Eaux et Forêts of Madagascar and the Ministry of Agriculture, Forestry and Fisheries in Cambodia. A total of 530 bats, belonging to seven families (Pteropodidae, Rhinolophidae, Hipposideridae, Megadermatidae, Emballonuridae, Vespertilionidae, and Molossidae) were trapped in the wild. A small sample of blood was obtained and the host was released without injury or in some cases retained as a voucher specimen.

### Microscopic examination

Thin blood smears were made for each animal. They were fixed in methanol and stained by incubation with 10% Giemsa for 10 minutes. The smears were examined by microscopy for haemosporidian parasites. Parasites isolated from bats were referred to as Haemosporidia sp. Positive blood smears were catalogued in the Département 'Régulations, Développement et Diversité Moléculaire', Muséum National d'Histoire Naturelle, Paris, France.

### DNA extraction and amplification

DNA was extracted from blood samples using the phenol/chloroform technique [12]. The 709 bp cytochrome *b *fragments were amplified using PCR and nested-PCR. The PCR reaction was carried out in a total volume of 25 μl under the following condition: 1 μl of each of the primers: PLAS 1 (5'-GAGAATTATGGAGTGGATGGTG-3') and PLAS 2 (5'-GTGGTAATTGACATCCWATCC-3'), 1 mM of each dNTP, 1 U of *Taq *polymerase (Solis), 3 mM MgCl_2_. The PCR conditions were: 5 min at 94°C, 30 sec at 94°C, 30 sec at 55°C and 1 min 30 sec at 72°C for 40 cycles and a final 10 min extension at 72°C. The nested-PCR was carried out using 1 μl of the PCR products and performed with the following primers: PLAS 3 (5'-GGTGTTTYAGATAYATGCAYGC-3') and PLAS 4 (5'-CATCCWATCCATARTAWAGCATAG-3'). The conditions were: 5 min at 94°C, 30 sec at 94°C, 30 sec at 55°C, 1 min 30 sec at 72°C for 40 cycles and a final 10 min extension at 72°C. The PCR products were sequenced using PLAS3 and PLAS4 primers by Macrogen (Korea). All of these primers were designed by the research group. They are specific to haemosporidian parasites and do not amplify others Apicomplexa parasites or host DNA. Relevant sequences were selected for the phylogenetic analysis. There are numerous avian parasite sequences available and some representative sequences were chosen based on geographical localizations.

### Phylogenetic analyses

The nucleotide sequences (709 bp) were translated into amino acid sequences to minimize homoplasy due to saturation of synonymous mutations since some taxa have diverged over hundreds of millions years. The sequences were aligned using CLUSTAL W [13]. Reference sequences of at least 219 amino acids without ambiguous positions were retrieved from GenBank. In the case of identical sequences in amino acids, only one sequence for the analysis was kept. Maximum Likelihood (ML) was performed using Phyml [14] and Parsimony analysis (P) was performed using Phylip package (version 3.62) [15]. Nodal robustness was evaluated by non-parametric bootstrap (100 replicates). Bayesian analyses were conducted with MrBayes V3.1 software [16] using gamma distribution, 1 000 000 generations (average standard deviation of split sequences is below 0.01), with tree sampling every 100 generations and a burn-in of 2500 trees. The aim of the study was to analyse Haemosporidia phylogeny, so *Theileria annulata*, *Babesia gibsoni *and *Toxoplasma gondii *which belong to the phylum Apicomplexa but are non-haemosporidian parasites were choose as out-groups. The phylogenetic study was run using cytochrome *b *sequences. This is the most abundant marker in GenBank for a variety of haemosporidian parasites and widely used in phylogenetic studies because reference sequences from other genes are lacking.

## Results

**Figure 1:**
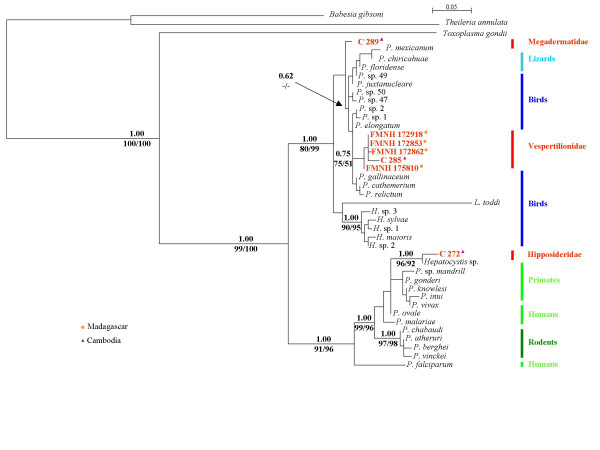
Phylogeny of Haemosporidia inferred from cytochrome *b *amino acid sequences. Value above branches are Bayesian posterior probabilities [16] (value less then 0.5 not shown), below are bootstrap percentage obtained by maximum likelihood [14] (left of the slash, values under 50% not shown). In red are the previously unpublished bat sequences. See Tables 1 and 2 for sampling details. *H*. = *Haemoproteus*, *L*. = *Leucocytozoon *and *P*. = *Plasmodium*.

530 blood samples were collected in Madagascar and in Cambodia belonging to seven families of bats. Haemosporidian infections were identified in three families: Hipposideridae, Vespertilionidae and Megadermatidae (Table 1 and Table 2). All the cytochrome *b *sequences that were isolated from these bats were previously unpublished and the parasite taxa, the host names, the collection localities and the GenBank accession numbers are given in Table 3. The three phylogenetic methods used, Parsimony (P), Maximum Likelihood (ML) and Bayesian analyses produced the same tree topology. The phylogeny is presented in Figure 1.

**Table 1:** Prevalence of Haemosporidia infection in bats from Madagascar

**Species**	**Family**	**n sampled**	**n infected (%)**
Eidolon dupreanum*	Pteropodidae	3	0
Rousettus madagascariensis*	Pteropodidae	33	0
*Hipposideros commersoni*	Hipposideridae	23	0
Triaenops furculus*	**Hipposideridae**	**28**	**1 (4)**
Triaenops rufus*	Hipposideridae	41	0
Emballonura tiavato*	Emballonuridae	2	0
***Miniopterus gleni***	**Vespertilionidae**	**13**	**3 (23)**
***Myotis goudoti***	**Vespertilionidae**	**68**	**16 (24)**
Miniopterus manavi*	**Vespertilionidae**	**129**	**49 (38)**
*Chaerephon *sp.*	Molossidae	1	0
Mormoproteus jugularis*	Molossidae	25	0
Otomops madagascariensis*	Molossidae	23	0
Chaerephon leucogaster*	Molossidae	9	0
*Mops leucostigma*	Molossidae	42	0

**Total**		**440**	**69**

* endemic species

**Table 2:** Prevalence of Haemosporidia infection in bats from Cambodia

**Species**	**Family**	**n sampled**	**n infected (%)**
*Rhinolophus acuminatus*	Rhinolophidae	3	0
*Rhinolophus borneensis*	Rhinolophidae	3	0
*Rhinolophus malayanus*	Rhinolophidae	1	0
*Rhinolophus shameli*	Rhinolophidae	2	0
*Rhinolophus *sp.	Rhinolophidae	1	0
*Hipposideros armiger*	Hipposideridae	2	0
***Hipposideros larvatus***	**Hipposideridae**	**13**	**1 (8)**
*Hipposideros pomona*	Hipposideridae	1	0
***Megaderma spasma***	**Megadermatidae**	**5**	**4 (80)**
*Taphozous melanopogon*	Emballonuridae	19	0
*Glischropus *sp.	Vespertilionidae	3	0
*Murina cyclotis*	Vespertilionidae	4	0
*Murina tubinaris*	Vespertilionidae	6	0
*Myotis muricola*	Vespertilionidae	1	0
***Kerivoula hardwickii***	**Vespertilionidae**	**10**	**2 (20)**
*Kerivoula papillosa*	Vespertilionidae	4	0
*Tylonycteris robustula*	Vespertilionidae	7	0
*Chaerephon plicatus*	Molossidae	5	0

**Total**		**90**	**7**

**Table 3:** Parasite taxa used in this study with host name, geographic location and GenBank accession number of the sequences used for the phylogenetic analysis

**Parasites**	**Host**	**Geographic locality**	**GenBank accession number**
*Plasmodium falciparum*	*Homo sapiens*	Tropical regions	AY069605
*Plasmodium gonderi*	Old world monkeys	Central Africa	AF069622
*Plasmodium inui*	Old world monkeys	India and southeast Asia	AF069617
*Plasmodium knowlesi*	Old world monkeys	Malaysia	AF069621
*Plasmodium malariae*	*Homo sapiens*	Tropical and subtropical regions	AF069624
*Plasmodium ovale*	*Homo sapiens*	Tropics of Africa and Asia	AF069625
*Plasmodium vivax*	*Homo sapiens*	Tropical and subtropical regions	AF069619
*Hepatocystis *sp.	*Papio *sp.	Ethiopia	AF069626
*Plasmodium *sp.	*Mandrillus leucophaeus*	Gabon	AF069623
*Plasmodium atheruri*	*Atherurus africanus*	Congo and Cameroon	AY099054
*Plasmodium berghei*	*Grammomys surdaster*	Central Africa	AF099049
*Plasmodium chabaudi*	*Thamnomys rutilans*	Central Africa	AF099050
*Plasmodium vinckei*	*Grammomys surdaster*	Congo	AY099052
*Plasmodium elongatum*	*Passer domesticus*	North America	AF069611
*Plasmodium gallinaceum*	*Gallus gallus*	Vietnam	DQ212189
*Plasmodium relictum*	*Zeneida macroura*	North America	AY099032
*Plasmodium *sp. 2	*Ninox scutulata*	Singapore	AY099035
*Plasmodium *sp. 1	*Acrocephalus arundinaceus*	Japan	AY099044
*Plasmodium juxtanucleare*	*Gallus gallus*	Asia	AB250415
*Plasmodium cathemerium*	Wide range Bird	Wallacean zones	AY377128
*Plasmodium *sp. 47	*Agelaius phoeniceus*	North America	AF465547
*Plasmodium *sp. 49	*Zonotrichia leucophrys*	North America	AF465549
*Plasmodium *sp. 50	*Andropadus latirostris*	Cameroon	AF465550
*Plasmodium floridense*	*Anolis oculatus*	Dominica	AY099059
*Plasmodium mexicanum*	*Sceloporus occidentalis*	California	AY099060
*Plasmodium chiricahuae*	*Sceloporus jarrovi*	Arizona	AY099061
Haemosporidia (FMNH 172853)	*Miniopterus manavi*	Madagascar	AY762070*
Haemosporidia (FMNH 172862)	*Miniopterus manavi*	Madagascar	AY762071*
Haemosporidia (FMNH 172918)	*Miniopterus manavi*	Madagascar	AY762074*
Haemosporidia (FMNH 175810)	*Myotis goudoti*	Madagascar	AY762075*
Haemosporidia (C285)	*Kerivoula hardwickii*	Cambodia	EF179354*
Haemosporidia (C289)	*Megaderma spasma*	Cambodia	EF179355*
Haemosporidia (C272)	*Hipposideros larvatus*	Cambodia	EF179356*
*Haemoproteus majoris*	*Parus caeruleus*	Sweden	AY099045
*Haemoproteus sylvae*	*Acrocephalus arundinaceus*	Sweden	AY099040
*Haemoproteus *sp. 1	*Phylloscopus occipitalis*	India	AY099043
*Haemoproteus *sp. 2	*Phylloscopus occipitalis*	India	AY099043
*Haemoproteus *sp. 3	*Acrocephalus scirpaceus*	Spain	AY099046
*Leucocytozoon toddi*	*Accipiter francesii*	Madagascar	AY684973
*Toxoplasma gondii*			AF023246
*Theileria annulata*			M63015
*Babesia gibsoni*			AB215096

FMNH: Field Museum of Natural History

C: Cambodia

* Previously unpublished sequences

**Figure 2:**
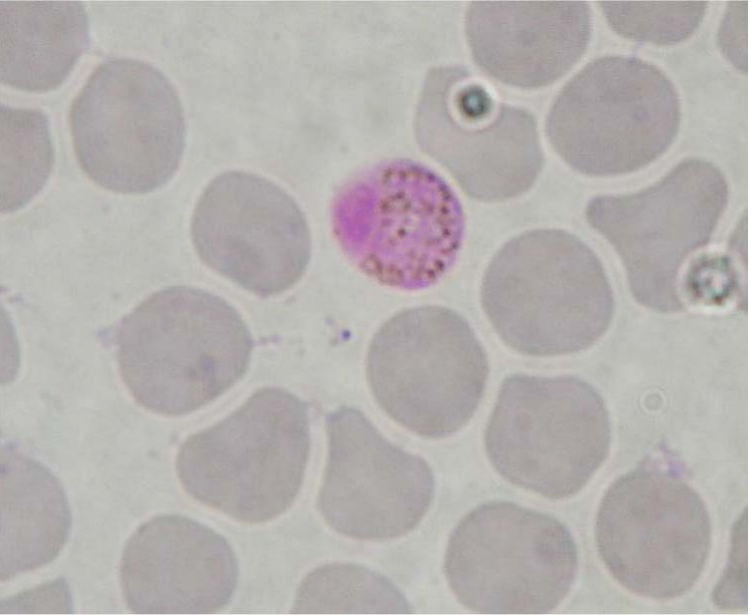
Haemosporidian parasite isolated from *Hipposideros larvatus *(C 272).

**Figure 3:**
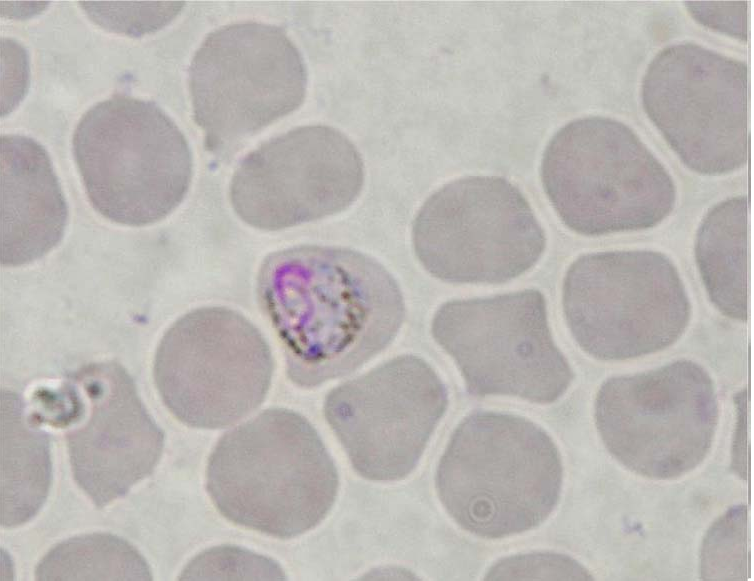
Haemosporidian parasite isolated from *Hipposideros larvatus *(C 272).

The results show the existence of two clades within Haemosporidia, separating mammal and sauropsid hosts (birds and lizards) (1.00 Bayesian posterior probabilities, 99 and 100 for ML and P respectively bootstrap support). In the first clade, the four malaria parasites afflicting humans, *Plasmodium malariae, Plasmodium ovale Plasmodium vivax *and *P. falciparum *form a polyphyletic group [17]. Rodent *Plasmodium *are the sister group and *P. falciparum *still exhibits a deep branch. Interestingly, parasite isolated from the bat *Hipposideros larvatus *(Family Hipposideridae) (Figures 2 and 3) clusters with a *Hepatocystis *parasite obtained from a baboon (*Papio s*p.) and falls within the *Plasmodium *primate group (Bayesian posterior probabilities of 1.00 and bootstrap support of 99 and 96 for ML and P, respectively).

**Figure 4:**
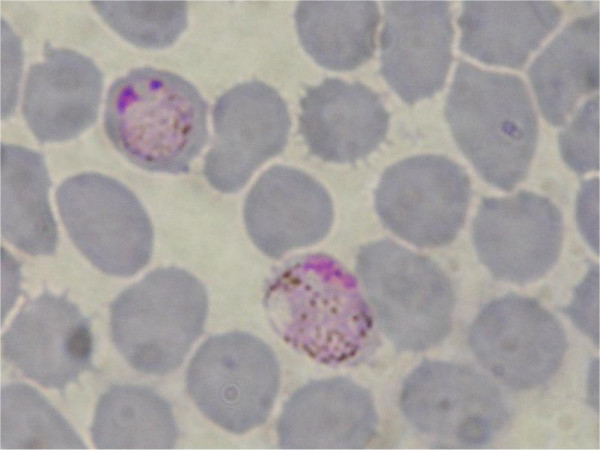
Haemosporidian parasites isolated from *Megaderma spasma *(C 289).

**Figure 5:**
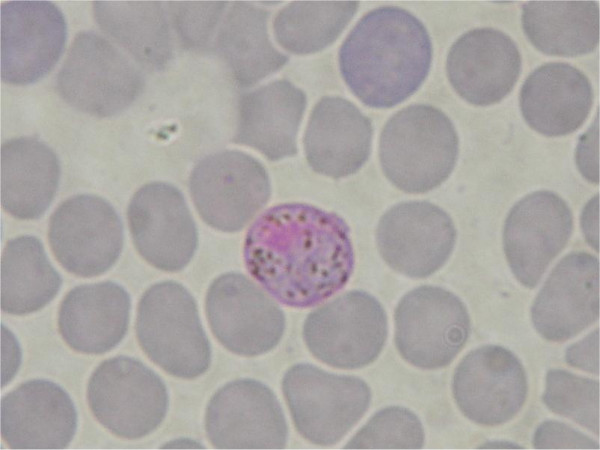
Haemosporidian parasite isolated from *Kerivoula hardwickii *(C 285).

In the second clade, between two clades of *Plasmodium*, are *Leucocytozoon *and *Haemoproteus*, two genera infecting only sauropsid hosts, and are close sister taxa of bird and lizard *Plasmodium *[18]. This renders the genus *Plasmodium *polyphyletic. Unexpectedly, the haemosporidian parasite isolated from the bat *Megaderma spasma *(Family Megadermatidae) (Figure 4) is included within the sauropsid *Plasmodium *clade. However, the bootstrap value is low (Bayesian posterior probabilities of 0.62). Remarkably, haemosporidian parasites isolated from the Malagasy endemic bats *Myotis goudoti *and *Miniopterus manavi *and the parasite isolated from the widespread Asiatic bat *Kerivoula hardwickii *(Figure 5) form a monophyletic cluster and fall within the sauropsid *Plasmodium *clade. All these bats are classically placed in the Family Vespertilionidae. This result is well support with 0.75 Bayesian posterior probabilities and 75 ML bootstrap.

## Discussion

The mitochondrial genome is more conserved in apicomplexan parasites [19] than in others metazoan eukaryotes. Cytochrome *b *gene is, therefore, a good marker to establish phylogenetic relationships between parasites that diverged several millions years ago [18].

The presence of a major division within haemosporidian parasites separating mammal and sauropsid hosts, suggests that parasites of these two vertebrate groups evolved separately. In the mammalian clade of parasites, except for *P. falciparum*, all primate *Plasmodium *species share a common ancestor, although host shifts occurred during the course of primate speciation. Indeed, wild primate populations are potential reservoirs for human malaria parasites [8]and host shifts have occurred in Southeast Asia with, for example, *Plasmodium knowlesi*, which usually infect macaques, afflicting humans [20]. Recently evidence on the origin of *P. vivax *as a macaque monkey malaria parasite in Southeast Asia has been proposed [7,21]. In addition, *Plasmodium simium *and *Plasmodium brasilianum*, two species infecting South American platyrrhini primates, are genetically indistinguishable from *P. vivax *and *P. malariae*, respectively, and may have been associated with a human-platyrrhini host-switch [8,21]. Further, haemosporidian parasite isolated from *Hipposideros larvatus *clusters with a baboon *Hepatocystis*. This association between bat and primate parasites has been previously proposed based on mitochondrial data, where a *Hepatocystis *species isolated from a bat (*Cynopterus brachyotis*, Family Pteropodidae) clusters with this baboon *Hepatocystis *[4]. Needless to say, this result is not congruent with mammal phylogeny [22] and suggests a host switch from primates to bats.

In the sauropsid clade, the evolutionary history of *Plasmodium *that infects birds and lizards is not resolved. The parasite phylogeny clearly does not fit with the host phylogeny. *Plasmodium *parasites from birds and lizards are known to show little host specificity [23]. Previous conclusions support that infrequent and unpredictable host shifts have occurred in the parasite-host sauropsid system [24]. Surprisingly, the Haemosporidia isolated from *Megaderma spasma *in Cambodia falls within the sauropsid *Plasmodium *clade. However, phylogenetic relationships between the parasites of *Megaderma *and sauropsids are not completely resolved.

Furthermore, closely related haemosporidian parasites isolated from *Myotis goudoti *and *Miniopterus manavi*, two endemic Malagasy bat species and the haemosporidian parasite from the Cambodian *Kerivoula hardwickii*, all of which are placed in the family Vespertilionidae, fall within the sauropsid *Plasmodium *clade. This result clearly does not fit with vertebrate phylogeny and supports host switching from birds to bats. The haemosporidian vespertilionid parasites from Madagascar and Cambodia are monophyletic, which suggest that the host switching took place in the early evolutionary history of these bats and was followed by subsequent radiation and co-speciation. This is the first report showing host switching in haemosporidian parasites between birds and mammals (bats).

After rodents, bats are the largest order of mammals (at least 1,100 species, more than 20% of extant mammal species). The Chiroptera are very diverse and they are distributed almost worldwide and have extremely diverse life history traits and morphology. Based on recent molecular work, they are classified into four super-families that apparently diversified in different areas during the early Eocene as a "Big Bang" radiation [25] coincident with the peak of Tertiary insect diversity [26]. In developing echolocation and different flight strategies, the ancestors of modern bats colonized various ecological niches [27], where birds and their associated blood parasites are thought to have been present, thus favoring host switching from birds to bats. Furthermore, *Myotis goudoti *and *Miniopterus manavi *often share common day roost sites in tree hollows, caves and rock shelters [28], which expose considerable numbers of densely packed individuals to the same potential blood parasite vectors.

## Conclusion

The introduction of the 7 new genetic sequences from chiropteran hemoparasites does not alter the deep branching of *P. falciparum *within the mammalian clade [4,17]. This result does not support a recent parasite transfer from birds to *Homo sapiens*, which has been used to explain the pathogenicity of *P. falciparum *in humans. Rather, the nearly exponential recent growth in human populations may have acted on *P. falciparum *selection patterns [29]. However, the sequence data from blood parasites isolated from bats provide further insights into the possible evolutionary pathway of human malaria parasites. Those results show that *P. falciparum *has a different and independent evolutionary history than other human malaria parasites. This is consistent with recent studies providing genetic evidence that the four human parasites did not emerge from the same geographical region [30]. Based on clear evidence presented herein of host switching between birds and bats, it is difficult to reject the hypothesis that *P. falciparum *has a non-primate origin. It may have emerged in humans associated with an ancient host switching and, as such, it could be one of the oldest "emerging" diseases in humans.

## Authors' contributions

LD, VR, and FA designed the study. LD, VR, GC, MR, JW, TN, SMG and FA do the sampling, LD and TN process samples and LD, HA, VR, SMG and FA analysed the data. LD wrote the first draft of the manuscript then VR, SMG and FA critically reviewed the manuscript. All authors read and approved the final manuscript.
